# Molecular characterization and heterogeneity of circulating tumor cells in breast cancer

**DOI:** 10.1007/s10549-017-4452-9

**Published:** 2017-08-16

**Authors:** Anna Jakabova, Zuzana Bielcikova, Eliska Pospisilova, Rafal Matkowski, Bartlomiej Szynglarewicz, Urszula Staszek-Szewczyk, Milada Zemanova, Lubos Petruzelka, Petra Eliasova, Katarina Kolostova, Vladimir Bobek

**Affiliations:** 1 Department of Laboratory Genetics, University Hospital Kralovske Vinohrady, Srobarova 50, 100 34 Prague, Czech Republic; 2 Department of Oncology, First Faculty of Medicine, Charles University and General University Hospital in Prague, Prague, Czech Republic; 3 Division of Surgical Oncology and Department of Oncology, Wroclaw Medical University, Wybrzeże Ludwika Pasteura 1, 50-367 Wrocław, Poland; 4 Breast Unit, Lower Silesian Cancer Center, Wroclaw, Plac Hirszfelda 12, 53-413, Wrocław, Poland; 5 Department of Surgery, University Hospital Motol, V Uvalu 84, 150 06 Prague, Czech Republic; 6 Faculty of Medicine, Charles University, V Uvalu 84, 150 06 Prague, Czech Republic; 7 Department of Thoracic Surgery, Masaryk´s Hospital, Krajska zdravotni a.s., Socialni pece 3316/12A, 40113 Usti Nad Labem, Czech Republic; 8 Department of Histology and Embryology, Wroclaw Medical University, Wybrzeże Ludwika Pasteura 1, 50-367 Wrocław, Poland

**Keywords:** CTCs, Circulating tumor cells, Breast cancer, Cultivation, In vitro, MetaCell, Gene expression

## Abstract

**Introduction:**

This study analyzes peripheral blood samples from breast cancer (BC) patients. CTCs from peripheral blood were enriched by size-based separation and were then cultivated in vitro. The primary aim of this study was to demonstrate the antigen independent CTC separation method with high CTC recovery. Subsequently, CTCs enriched several times during the treatment were characterized molecularly.

**Methods:**

Patients with different stages of BC (*N* = 167) were included into the study. All patients were candidates for surgery, surgical diagnostics, or were undergoing chemotherapy. In parallel, 20 patients were monitored regularly and in addition to CTC presence, also CTC character was examined by qPCR, with special focus on HER2 and ESR status.

**Results:**

CTC positivity in the cohort was 76%. There was no significant difference between the tested groups, but the highest CTC occurrence was identified in the group undergoing surgery and similarly in the group before the start of neoadjuvant treatment. On the other hand, the lowest CTC frequencies were observed in the menopausal patient group (56%), ESR+ patient group (60%), and DCIS group (44.4%). It is worth noting that after completion of neoadjuvant therapy (NACT) CTCs were present in 77.7% of cases. On the other hand, patients under hormonal treatment were CTC positive only in 52% of cases.

**Discussions:**

Interestingly, HER2 and ESR status of CTCs differs from the status of primary tumor. In 50% of patients HER2 status on CTCs changed not only from HER2+ to HER2−, but also from HER2– to HER2+ (33%). ESR status in CTCs changed only in one direction from ESR+ to ESR−.

**Conclusions:**

Data obtained from the present study suggest that BC is a heterogeneous disease but CTCs may be detected independently of the disease characteristics in 76% of patients at any time point during the course of the disease. This relatively high CTC occurrence in BC should be considered when planning the long-term patient monitoring.

## Introduction

Enumeration of circulating tumor cells (CTCs) has showed a prognostic role in various stages of the breast cancer (BC). Hormone receptors (estrogen and progesterone) and HER2 status of primary BC tumor have been established during standard clinical biopsies and are of crucial importance in the choice of treatment. Real-time tumor monitoring through CTC enumeration could be an important indicator of individual cancer development [1].

CTCs as biomarkers can offer some valuable information about a patient’s tumor, if detection, separation, and characterization are performed in a reliable manner. Although occurrence of CTCs in patients’ peripheral blood is often very low, enrichment methods can be introduced for CTC separation before their characterization. They are usually based on surface protein expression, size, density, electric charges, or deformability of CTCs.

This study analyzes peripheral blood samples from patients with BC. CTCs from peripheral blood were enriched by size-based separation and then cultivated in vitro. The primary aim of this study was to demonstrate the antigen independent high sensitive separation method and a possibility of molecular characterization of CTCs enriched several times during the treatment.

## Materials and methods

### Patients

To date 167 patients with diagnosed BC have been enrolled in the study in accordance with the Declaration of Helsinki. All patients were candidates for surgery, surgical diagnostics, or with planned or applied chemotherapy. Based on their informed consent, clinical data were collected from all participating patients. Basic cytopathological data are reported in Table 1. For each patient, approximately 2 × 8 mL of venous blood was drawn from the antecubital veins and placed into S-Monovette tubes (Sarstedt AG & Co., Numbrecht, Germany) containing 1.6 mg EDTA/mL blood as an anticoagulant. The samples were processed at room temperature using an isolation procedure completed within 24 h after the blood draw.

**Table 1:** Basic cytopathological characteristics of patients

	*N*	(%)
Stage		
0	3	2
IA	45	30
IIA	64	42.7
IIB	20	13.3
IIIA	13	8.7
IIIB	1	0.67
IIIC	4	2.67
Histopathological features		
Benign	2	1.7
DCIS	9	7.6
LCIS	1	0.85
IDC (NST)	76	65.6
ILC	14	11.86
Mixed	16	13.6
Menopausal status		
Premenopausal	65	39.39
Menopausal	18	10.9
Postmenopausal	82	49.7
Tumor size		
T1	63	61.1
T2	36	34.9
T3	4	3.8
Nodal involvement		
N0	56	56.5
N1	37	37.3
N2	6	6
Grading		
G1	7	11.8
G2	24	40.6
G3	28	47.4
HR and HER2 status		
HR+ HER2+	16	11.7
HR− HER2+	7	5.1
HR+ HER2−	91	66.4
HR− HER2−	23	16.8

### CTCs enrichment and culture

The recently introduced size-based separation method for viable CTC enrichment process (MetaCell^®^, MetaCell s.r.o., Ostrava, Czech Republic) [2–6] is based on the filtration of peripheral blood through a porous polycarbonate membrane (with pores of 8 μm diameter). The minimum and maximum volume of the filtered peripheral blood may be adjusted up to 50 mL of fluid. The standard 8 mL peripheral blood sample from patients suffering from BC was transferred into the filtration tube. Gradual transfer of the blood in several steps is preferred to prevent blood clotting on the membrane filter. The peripheral blood flow is supported by capillary action of the absorbent touching the membrane filter. The filtered CTCs were observed immediately after filtration on the membrane. The control and presence of filtered CTCs immediately after isolation eliminates false negative results. The membrane filter is kept in a plastic ring that is transferred into the 6-well cultivation plate, 4 mL RPMI media is added to the filter top and CTCs are cultured on the membrane in vitro under standard cell-culture conditions (37 °C, 5% atmospheric CO_2_) and observed by inverted microscope. The CTCs were grown in FBS-enriched RPMI medium (10%) for a minimum of 14 days on the membrane. Alternatively, the enriched CTC fraction can be transferred from the membrane and cultured directly on any plastic surface or a microscopic slide, or the separation membrane may be translocated on a microscopic slide. Microscopic slide is preferred if immunohistochemistry/immunofluorescence analysis is planned. If an immediate CTC analysis is awaited, the CTC fraction is transferred in PBS (1.5 mL) to a cytospin slide. The slide is then dried for 24 h and analyzed by histochemistry (May-Grünwald staining) and/or by automated immunohistochemistry protocols (Ventana, Benchmark Ultra, Roche) using standard differential diagnostic antibodies in the pathological evaluation process.

### Cytomorphological analysis

**T**he stained fixed cells captured on the membrane were examined using light microscopy in two steps: (i) screening at ×20 magnification to locate the cells; (ii) observation at ×40/×60 magnification for detailed cytomorphological analysis. Isolated cells and/or clusters of cells of interest (immunostained or not) were selected, digitized, and the images were then examined by an experienced researcher and/or pathologist. CTCs were defined as cells with the following characteristics: (i) with a nuclear size ≥10 μm); (ii) irregular nuclear contour; (iii) visible cytoplasm, cells size over 15 μm; (iv) prominent nucleoli; (v) high nuclear-cytoplasmic ratio; (vi) proliferation; (vii) actively invading cells creating 2D or 3D cell groups.

### Gene expression analysis (GEA)

The key purpose of GEA was to compare gene expression of tumor-associated markers in the CTC-enriched fractions to that in the whole blood (white blood cells). Gene expression analysis can be performed to confirm the origin of the captured cells on the separation membrane. Gene expression analysis (GEA) allows up to 20 tumor-associated markers in RNA from different cell fractions to be tested within a single quantitative polymerase chain reaction (qPCR) run. Differential diagnostics markers for qPCR test are chosen in accordance with the expected diagnosis.

RNA is isolated from the whole blood and CTC-enriched fraction on the membrane. The CTC-enriched fraction of cells grown on the separation membrane in vitro (the so-called “membrane fraction”) was used for RNA isolation.

Finally, CTC-gene expression analysis allows identification of the relative amount of tumor-associated (TA) markers in the whole blood and in CTC-enriched fractions. If the tumor-associated genes are highly expressed in the CTC fraction, a subsequent analysis of chemoresistance-associated (CA) genes is performed. Molecular analysis helps to identify which type of chemotherapeutic agents may be of use in tumor therapy and assigned as personalized cancer therapy based on CTC.

The cells captured on the membrane are lysed by RLT-buffer with beta-mercapto-ethanol (Qiagen). RNA is then isolated using the RNeasy Mini Kit (Qiagen). RNA from the whole blood is isolated with a modified procedure and the quality/concentration of RNA is measured by NanoDrop (ThermoScientific). As there are only up to a few hundred cells on the membrane, the median concentration of RNA is quite low (5–10 ng/µl). High Capacity cDNA Reverse Transcription Kit (Life Technologies) was used for cDNA production. Gene expression analysis was performed using Taqman chemistry with Taqman MGB-probes for all the tested genes (Life Technologies).

The following genes associated with tumorigenic character and therapeutic potential in breast cancer were chosen for the multimarker GEA panel: ACTIN, CD45, CD68, EPCAM, MUC1, KRT18, KRT19, ESR, PGR, MAMMAGLOBIN, HER2, CD24, CD44. Additionally, genes associated with chemoresistance were tested (MRP1-10, MDR1, ERCC1).

### Statistical analysis

All analyses were performed using clinicopathological information transformed into variables 0 and 1 if applicable for tested characteristics. Chi squared test, *t* tests, cluster analysis, and correlation analysis of qPCR data were outperformed using GeneX (MultiD, SE) and GraphPadPrism versus 5 (Graphpad, US). *P* value of less than 0.05 was considered statistically significant.

## Results

**Fig. 1:**
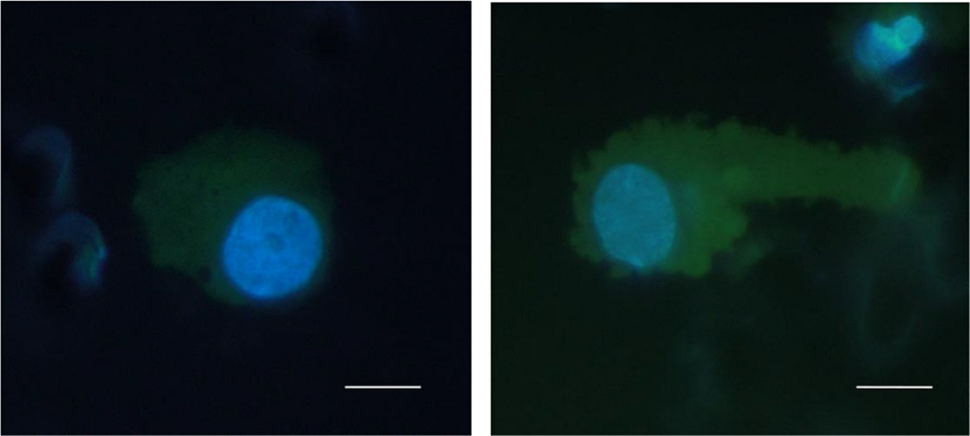
CTCs isolated from a patient with breast cancer, captured on the separation membrane (vital fluorescent staining— NucBlue^®^ and Celltracker^®^). *Bar* represents 10 µm

The main focus of the study was to detect CTCs shortage in BC patients by a new methodological approach which is based on size-dependent separation of CTCs and subsequent cytomorphological evaluation. Cytomorphological evaluation using vital fluorescence microscopy approach (Fig. 1) enables further use of the viable captured cells for RNA/DNA analysis.

Patients diagnosed with different stages of breast cancer (BC) (*N* = 167) were included into the study. The patients were divided based on clinicopathological criteria and CTC presence was tested. Summary of the collected CTC positivity data is presented in Table 2.

**Table 2:** CTC positivity identified in BC—patient subgroups

	*N*	(%)
CTC Positivity	CTC+	
CTC+	**119**	**72.1**
CTC−	**46**	**27.9**
Stage		
0	3	100
IA	31	68.9
IIA	47	73.4
IIB	16	80
IIIA	10	76.9
IIIB	0	0
IIIC	4	100
Histopathological features		
DCIS	4	**44.4**
LCIS	1	100
IDC (NST)	55	72.4
ILC	13	92.9
Mixed	9	56.3
Menopausal status		
Premenopausal	51	78.4
Menopausal	10	**55.5**
Postmenopausal	58	70.7
Tumor size		
T1	63	74.6
T2	36	88.8
T3	4	75
Nodal involvement		
N0	23	82
N1	17	78
N2	2	66
Grading		
G1	**5**	70
G2	14	58
G3	23	82
HR and HER2 status		
HR+ HER2+	13	81.3
HR− HER2+	7	100
HR+ HER2−	64	**70.3**
HR− HER2−	22	95.7
ESR+ PGR+ vs. ESR+ PGR−		
Therapy		
Before therapy	28	82.3
During HT	9	**52.9**
After NACT	7	77.7
Before surgery (after biopsy)	39	86.6

CTC positivity in tested cohort was 76%. There was no significant difference between tested subgroups, identifying a possible CTC presence, but the highest CTC occurrence was observed in the group undergoing surgery (86.6%) and similarly in the group before the start of neoadjuvant and adjuvant treatment (82.3%).

It is important to comment on relatively high CTCs presence even after neoadjuvant therapy has been completed (77.7%). It can be assumed that in these patients therapy did not diminished all the cancer cell types.

There were no significant differences in CTC frequencies observed based on stage definitions. Considering the histopathological character of the primary tumor, the lowest CTC positivity was observed in DCIS (44.4%). Relatively low CTC frequency was observed in the menopausal patient group (55.5%).

**Fig. 2:**
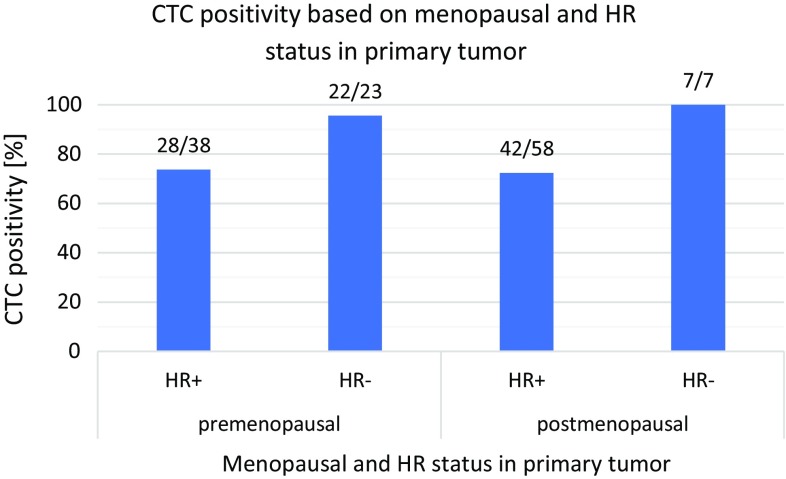
CTC positivity in relation to menopausal stage and primary tumor HR− expression

Furthermore, it can be concluded that in tumors with ESR expression (ESR+) and without PGR expression (PGR-) CTCs were detected only in 60% (9/15) of tested cases, whereas in ESR+/PGR+ tumors CTC positivity was 73% (68/93). On the other hand, in patients with ESR- negative tumors CTCs were detected in 96.7% which is almost all of the patients under study (30/31). Therefore, it must be mentioned that during the therapy only 52.9% (9/17) of patients exhibited CTCs. Nevertheless, menopausal stage has to be considered if ESR/PGR expression is evaluated. The correlation of the menopausal status and ESR/PGR expression is illustrated in Fig. 2 which shows that hormonal receptor-positive tumors exhibit the lowest CTC detection frequencies in comparison to the HR- groups.

**Fig. 3:**
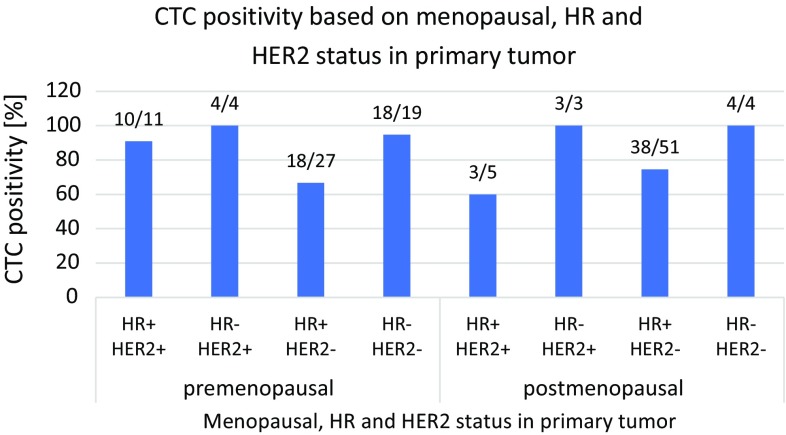
CTC positivity in relation to menopausal stage and primary tumor HR and HER2− expression

Similarly, even if not statistically significant, it can be seen that HR +/HER2− tumors, irrespective of the menopausal stage show the lowest CTC frequency rates (see Fig. 3).

In parallel, 20 patients were monitored regularly during the course of the disease and in addition to CTC presence, CTCs character was also examined by qPCR with special focus on HER2 and ESR status. In total 43 qPCR analysis were evaluated. Therapeutically, the most relevant findings are as follows: HER2 and ESR status of CTCs may differ from the status of primary tumor.

The most frequent changes were seen in the triple negative BC (TNBC) group (*N* = 12) where 27 samples were evaluated. HER2 presence was confirmed in CTCs in four cases, which means that the change from HER2– to HER2+ occurred in 15% of tested samples, but that these four changes can be ascribed to four different patients. The change was relevant for four out of 12 patients (33.3%) which is already a significant number. Similarly, in 50% of patients, HER2 status changed from HER2+ to HER2− (3/6).

ESR status in CTCs changed only in one direction from ESR+ to ESR− (3/3). These patients’ primary tumors were diagnosed as ESR+/PGR+/HER2−. This group of patients will most probably exhibit very frequent changes.

Taken together, due to relatively high numbers of CTC positivity in different patient groups, we may conclude that a certain number of CTCs are always present in the blood of the patients. The cells have to be under selection pressure of treatment uninterruptedly. As soon as the selection pressure is stopped, new gene expression profile is displayed by CTCs.

The data obtained in the present study suggest that BC is a heterogeneous disease, but CTCs may be detected independently of the disease characteristics in 76% (119/165) of patients at any time point of the course of the disease. This relatively high CTC occurrence in BC should be considered in planning the long-term patient monitoring.

## Discussion

Treatment decisions in BC are based on the characteristics of the primary tumor without considering the character of minimal residual disease or metastasis. However, tumors are evolving entities and genetic heterogeneity has been detected comparing the primary tumor with subsequent recurrences and metastases and analyzing different regions of the same tumor [7]. It has been hypothesized that the success of personalized treatments greatly depends on the capability to capture and monitor tumor heterogeneity over time and to consequently modulate therapies [8].

Detection and characterization of CTCs can contribute to the understanding of the disease and improved therapy monitoring as well as personalized treatment options. The key step is sensitive isolation and detection of CTCs. To date, various approaches have been also used to visually identify CTCs; however, the techniques employed to perform cell enrichment, immunohistochemical detection, and image analysis are complicated [9, 10]. Moreover, epithelial markers are currently used to detect CTCs; tumor cells, however, may lose their epithelial features during metastasis/dissemination or may not express these markers because of their heterogeneity [11]. Therefore, some CTCs could be unidentified during epithelial-mesenchymal transition (EMT) by the common CTC-enrichment strategies relying on epithelial markers [12]. According to recent findings, more invasive CTCs may lose their epithelial antigens as a result of the EMT process [13] and EMT has been increasingly recognized as the key mechanism of cancer drug resistance [14].

We have used a simple method, without any complicated processing steps, for detecting viable human CTCs in the peripheral blood by using physical features of CTCs. We believe that viable CTCs may be a less invasive, repeatable biomarker for monitoring tumor responses.

In our study more than 76% patients were CTC positive. This result provides evidence that BC cells migrate and disseminate from morphologically very early lesions. Hosseini et al. demonstrated that metastatic dissemination often occurs early during tumor formation [15]. Disseminated cancer cells detected in patients before the manifestation of breast-cancer metastasis contain fewer genetic abnormalities than primary tumors and indicate that dissemination occurs during early stages of tumor growth [16–19].

As demonstrated by the SWOG S0500 trial, the simple enumeration of CTCs is not sufficient to guide therapy [20]. There is increasing evidence that cancer evolves over time because of its genomic instability and under the selection pressure of systemic treatments. These changes can be responsible for the appearance of drug-resistant clones. In studies of metastatic breast cancer (MBC), a discrepancy was observed between metastases or CTCs and the primary tumors in terms of HER2, estrogen and progesterone receptor expression [21, 22]. The loss of progesterone or estrogen hormone receptor expression in CTCs was described in 40% of receptor-positive MBC, while increased hormone receptor expression was detected in only 8% of triple negative MBC [21].

The clinical use of new CTC detection technique and the molecular characterization of isolated CTCs may lead to the development of personalized anticancer strategy in near future.
